# The Ecological View of Selective Attention

**DOI:** 10.3389/fnint.2022.856207

**Published:** 2022-03-21

**Authors:** Tidhar Lev-Ari, Hadar Beeri, Yoram Gutfreund

**Affiliations:** The Ruth and Bruce Rappaport Faculty of Medicine and Research Institute, The Technion, Haifa, Israel

**Keywords:** stimulus selection, evolution of attention, decision making, limited processing capacity, optic tectum

## Abstract

Accumulating evidence is supporting the hypothesis that our selective attention is a manifestation of mechanisms that evolved early in evolution and are shared by many organisms from different taxa. This surge of new data calls for the re-examination of our notions about attention, which have been dominated mostly by human psychology. Here, we present an hypothesis that challenges, based on evolutionary grounds, a common view of attention as a means to manage limited brain resources. We begin by arguing that evolutionary considerations do not favor the basic proposition of the limited brain resources view of attention, namely, that the capacity of the sensory organs to provide information exceeds the capacity of the brain to process this information. Moreover, physiological studies in animals and humans show that mechanisms of selective attention are highly demanding of brain resources, making it paradoxical to see attention as a means to release brain resources. Next, we build on the above arguments to address the question why attention evolved in evolution. We hypothesize that, to a certain extent, limiting sensory processing is adaptive irrespective of brain capacity. We call this hypothesis the ecological view of attention (EVA) because it is centered on interactions of an animal with its environment rather than on internal brain resources. In its essence is the notion that inherently noisy and degraded sensory inputs serve the animal’s adaptive, dynamic interactions with its environment. Attention primarily functions to resolve behavioral conflicts and false distractions. Hence, we evolved to focus on a particular target at the expense of others, not because of internal limitations, but to ensure that behavior is properly oriented and committed to its goals. Here, we expand on this notion and review evidence supporting it. We show how common results in human psychophysics and physiology can be reconciled with an EVA and discuss possible implications of the notion for interpreting current results and guiding future research.

## Introduction

Selective attention is a behavioral process by which a feature, event, object or location is prioritized over other simultaneous possibilities (Fiebelkorn and Kastner, 2020). It is by no doubt a fundamental process in our daily lives governing the way we explore, think and perceive the environment (Carrasco, 2011). As famously noted by Williams James, we are all very familiar with the many consequences of selective attention such as searching for a person with a red shirt in a crowd, focusing on the patch of skin in which a needle is about to enter, abruptly looking at (and thinking about) the location of a sudden sound in a dark alley. Attention is vital for normal behavior, with upsetting consequences when malfunctioning (Posner et al., 2020). Consequently, attention has been one of the most studied topics in neuroscience and psychology in the past 100 years, researched and discussed extensively. Yet, basic questions about attention are still debated such as: What is the evolutionary origin of attention? What is the purpose of having attention? What is being allocated and at what level? (Allport, 1993; Anderson, 2011). Among the different viewpoints about attention, one stands out to dominate the field. The essence of this view is the claim that our brain is bombarded by sensory information, which exceeds what its processing capacity can handle. Hence, attention is nature’s way to cope with this limitation by sequentially selecting bits and pieces of the incoming information for detailed processing. Below are examples of typical quotes:

*“Nowhere is this more evident than in the primate’s visual system, where the amount of information coming down the optic nerve — estimated to be on the order of 10^8^ bits per second — far exceeds what the brain is capable of fully processing and assimilating into conscious experience. The strategy nature has devised for dealing with this bottleneck is to select certain portions of the input to be processed preferentially, shifting the processing focus from one location to another in a serial fashion”*

(Itti and Koch, 2000).

*“The primate visual system has a limited information processing capacity*… *this limited capacity – visual attention – is dynamically allocated”*

(Albright et al., 2000).

*“The amount of visual information entering our eyes is much greater than what our brain can fully process. It is therefore necessary that we can select information that is relevant and ignore information that is irrelevant for our tasks”*

(Theeuwes et al., 2010).

These representative quotes illustrate how our thinking of selective attention is dominated by the notion of attention as a means to solve the problem of increased demand to process information by limited sensory processing resources in the brain. In this review, we refer to the above notion as the limited brain resources view of attention (LBRV).

While the LBRV has been a very influential and useful framework for thinking about attention, it has its limitations and raises some conceptual difficulties. To what extent is the brain capacity to process information low relative to the capacity of the sensory systems to provide information is an unresolved and debated question (Allport, 1989; van der Heijden and Bem, 1997b). In this opinion paper, we take an evolutionary approach to address this question. We ask: What is the driving force behind the evolutionary development of mechanisms of attention? Is it indeed the demand to process more information with a limited processing capacity or maybe the other way around; processing information is limited, at times, because the brain evolved to support mechanisms of attention? Based on evolutionary considerations, we suggest a view that favors the latter possibility. We call this view the ecological view of attention (EVA) because it is centered on the interaction between an animal and its environment, and, in several ways, is related to Gibson’s theory of ecological perception (Gibson, 2014). In its essence is the notion that the sole role of the sensory systems is to serve adaptive interactions of an animal with its environment. The incoming inputs are integrated with internal information and the outcome is adaptive behavior, with attention being part of it. The brain is built to create robust attention because otherwise it would have been behaviorally maladaptive. The EVA does not deny that there are limited cognitive capacities in the brain. However, it postulates that internal limited resources are not necessary for the evolution of attention.

**FIGURE 1:**
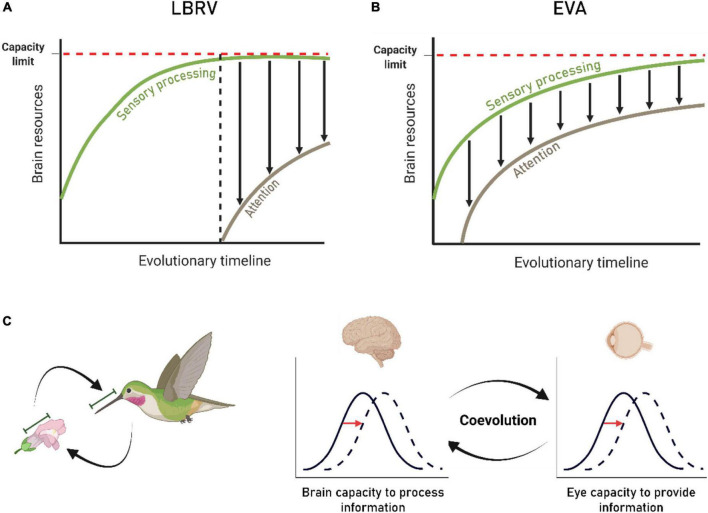
Evolutionary considerations about stimulus selection and brain capacity. **(A)** Evolutionary timeline of attention under the traditional view [limited brain resources view of attention (LBRV)]. The green curve depicts resources required for the function of the brain. The competition over food resources drives an evolutionary increase in brain size and complexity, up to an upper physical limit (red dash line). At this point, attention emerges as a solution to the limited capacity problem (pink curve). However, selection and suppression (i.e., attention) consumes brain resources. This implies that attention has to come with a cost of less resources available for sensory processing (black arrows lengths). **(B)** Under the ecological view of attention (EVA) scheme stimulus selection and suppression of distractors (i.e., attention) is an important, indistinguishable part of sensory processing, both evolving together (pink and green curves). The EVA does not deny a limited capacity (red dash line). However, it asserts that attention evolved independent of the brain reaching its limited capacity. **(C)** The principle of coevolution is illustrated by the perfect match that can be found between the beak shapes of certain bird species and their host flowers. This principle, similarly predicts a match between the eye’s capacity to provide information and the brain’s capacity to make use of this information for adaptive behavioral control. The reciprocal arrows indicate the selective pressures within a coevolving system.

The distinction between the two notions (LBRV and EVA) is significant. On the one hand, attention is viewed as a high-level cognitive capability that evolved in higher animals as a response to the increased demand for processing more information, while on the other hand, attention is viewed as a basic process in brain evolution that can be traced in all active food-seeking organisms (Figures 1A,B). Both notions require neuronal mechanisms to detect the most salient and important stimulus as well as mechanisms to suppress irrelevant stimuli. The basic argument is less about mechanisms of attention and more about the reasons for these mechanisms to evolve. Nevertheless, as we argue below, this should not make us underestimate the importance of the distinction for interpreting current results, for guiding future research and for influencing the ways we treat attentional disorders.

## Evolutionary Considerations Do Not Support the Limited Brain Resources View of Attention

The development of the LBRV is rooted to a large extent in human psychology (Treisman, 1964; Broadbent, 1965). Evolutionary and comparative considerations can lead to different insights (Krauzlis et al., 2018, 2021). Following are three evolutionary arguments that question the main notions of the LBRV.

1. Evolutionary considerations do not favor the claim that the information provided by the sensory organs exceeds the brain’s capacity to process the information. The central visual system evolved in size and complexity to process and use information from the eye, which, in turn, evolved to provide precise information to the brain. Just as brain processing of information is resource-demanding, so does acquiring and providing information by the eyes. Thus, the co-evolution of the eye and brain argues that the brain’s capacity to process information should limit the eye’s capacity to provide information, and vice versa (Garamszegi et al., 2002). This evolutionary loop entails that the capacity of the sensory organs to provide information should not exceed the capacity of the brain to process and make use of the information (Figure 1C), an evolutionary constraint that is inconsistent with the common claim that the brain is bombarded with sensory information, beyond what it can process.

2. The energetic/processing costs of selective attention may outweigh the gains. One important aspect of selective attention is that it is far from resembling a passive filter. Selection mechanisms work to identify the most behaviorally relevant event or object at any particular moment. Successful prioritization is critically important for the survival of an animal and it is clear why evolution favors mechanisms that lead to precise, fast, context and memory-dependent selection. However, such mechanisms consume resources. In dynamical, cluttered environments, the task of selecting the most appropriate stimulus is computationally demanding, requiring the integration of multiple information sources with internal states and memory (Moore and Zirnsak, 2017). Even a seemingly simple bottom-up task such as identifying a stimulus that breaks the regularity of its background (as in pop-out perception or deviance detection) is computationally difficult, needing neuronal networks to learn the statistical regularities of the background to compute differences from this regularity and to continuously update the internal model of the background (Gutfreund, 2012; Nelken, 2014). Indeed, brain networks for selective attention, which are gradually being exposed, are widespread, ranging from superior colliculus, thalamus, basal ganglia and a variety of neocortical areas (Bisley, 2011; Goll et al., 2015; Fiebelkorn and Kastner, 2020). All are critically involved in normal selective attention behaviors and are interconnected in a vast network. Micro-circuitry for stimulus selection within critical brain structures for attention such as the superior colliculus and lateral intraparietal area (area LIP) are precise and elaborate involving non-trivial and far-reaching lateral and recurrent interactions (Bisley and Goldberg, 2010; Mysore and Knudsen, 2011; Garrido-Charad et al., 2018). Thus, our ability to continuously select the most important stimulus and suppress the influence of distractors relies on vast computations and the exhaustion of major brain resources.

According to the LBRV, attentional mechanisms evolved to free brain resources for detailed processing of sensory information at the focus of attention (Navon and Gopher, 1979). However, as discussed above, attentional mechanisms by themselves involve considerable processing. Acquiring a process that by itself is a highly resourced consumer to free resources for a different process is evolutionary paradoxical. If an organism is limited in its ability to make use of the information from the senses and cannot increase brain size or complexity because of limitation of resources, due to the same limitations, this organism cannot develop mechanisms of attention.

3. The brain’s capacity to acquire and use vast amounts of information in parallel is outstanding. Mechanisms of selective attention seem to be robustly active even in simple detection tasks where the relevant information is minute (Posner et al., 1980). This poses a challenge to the LBRV. If the need to process vast information with a limited brain is the driving force behind the evolution of attention, why attention is at play when the capacity of information in the task is low? The human brain is an immensely complex information processing machine capable of processing multiple pieces of information in parallel, in detail and at high speed. Our bodily posture control system, for example, collects information from somatosensory, proprioceptors, vestibular and visual inputs, and processes all to control body posture with great accuracy and flexibility (Massion, 1994; Maurer et al., 2006). In the visual system, we process information effortlessly in parallel from wide and complex scenes and can identify objects of interest in less than 100 ms (Rousselet et al., 2004). In light of the high capacity of information processing by natural brains, why should there be a principal difficulty to process information simultaneously from two, five or more faces in a crowd? Why should there be a capacity limit in a simple two-alternative force choice cueing task to process simultaneously to the fullest two stimuli on both sides of the visual field? This, again, is evolutionary paradoxical: if the evolutionary drive behind attention is the economical need to allocate limited resources for information processing, it would seem maladaptive to allocate processing resources to one location at the expense of other locations in tasks for which the information capacity in the task is well below the limits of the brain, yet we do it (Pestilli and Carrasco, 2005).

## The Ancient Competition for Orientation and the Ecological View of Attention

The above arguments suggest that limited brain resources is not the evolutionary cause for the development of attention processes. What then is the evolutionary driving force behind attention? Mechanisms of stimulus selection are probably as old as the brain itself. The brain, the part of the nervous system within the head, evolved in parallel to the evolvement from radial symmetrical organisms that passively collect drifting food particles to bilateral symmetrical organisms that actively propel the body to bring the mouth to the food (Genikhovich and Technau, 2017). Consequently, the head that leads the body to its targets becomes home to the main sensory organs. In parallel, the brain, the ganglion within the head, increases in size to collect information from the main sensory organs to control the orientation of the head toward its targets (Sarnat and Netsky, 2002). However, for an active food-seeking organism to be successful it is not enough to just orient to a target; computational mechanisms should evolve to select the most appropriate target for behavior at any time as rapidly and efficiently as possible. In parallel, and not less important, once a target is selected, orienting behavior must be committed, therefore, mechanisms to suppress distraction of behavioral irrelevant signals should evolve. Thus, the two defining ingredients of attention – focusing on a selected target and withdrawing from others (Desimone and Duncan, 1995) – already emerge from the most basic behavioral interaction of the organism with its environment. We speculate that the competitive attempt to increase accuracy and flexibility of coordinated behaviors is the main evolutionary driving force behind the sophisticated context and memory-dependent selective attention processes that we are familiar with today.

However, the behavioral necessity to focus on a target and avoid distractors is a necessary but not sufficient requirement for attentional mechanisms. If the brain has the resources, it hypothetically can represent the whole visual field equally well, compute the distinction between the target and distractors and use this to guide behavior adaptively without attention. We argue that because the sensory organs dramatically subsample the information from the environment (Gibson, 2014) leading to sensory inputs that are ambiguous and noisy (Deneve, 2012), it is inherently difficult to distinguish targets from distractors (Eckstein, 2017). Attention mechanisms by-pass this difficulty by ensuring that once a target is selected, interference from distractors is suppressed, and thus behavior is maintained oriented and committed to its goal. Thus, we propose, that limited, noisy sensory inputs together with multiple behavioral opportunities are the dominant forces behind the evolution of selective attention.

*“Suppose two similar dates in front of a man, who has a strong desire for them but who is unable to take them both. Surely he will take one of them, through a quality in him, the nature of which is to differentiate between two similar things”*

(Kane, 2005).

This quote by 12th century Persian philosopher Abu Hamid al-Ghazali elegantly illustrates the merit of selective attention. The same notion is illustrated by the well-known Buridan’s ass paradox (Pessiglione and Clairis, 2019). A hungry donkey placed between equally appealing piles of hay will not starve to death in the middle because its brain possesses mechanisms that forces it to select and behaviorally commit to its decision. Attentional mechanisms, according to this notion, are nature’s way to prevent organisms from being stuck in a Buridan’s ass paradox.

**FIGURE 2:**
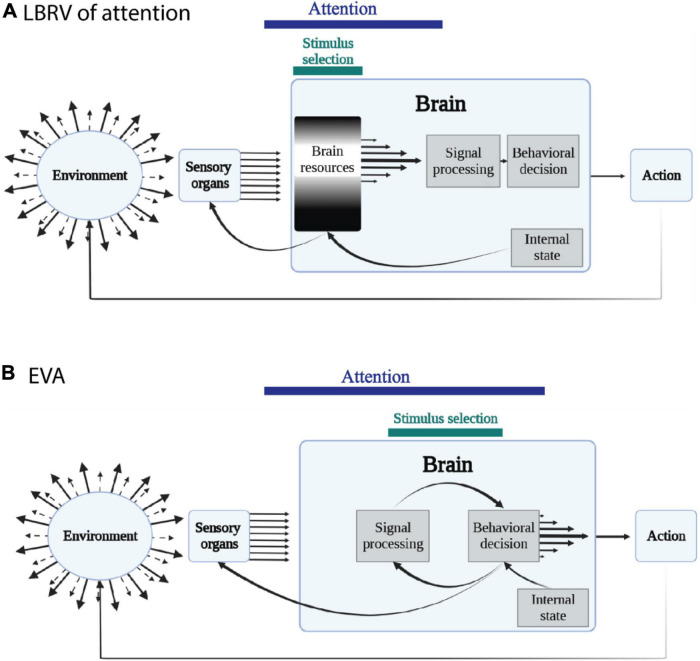
Comparison between the hypothesized environment-to-behavior pathways under the LBRV and under the EVA. **(A)** Under the classical LBRV of attention, the abundant and rich information in the environment is sub-sampled by the sensory organs and transmitted to the brain. In the brain, a limited resource (white shading) is allocated to process selected and filtered information for behavioral choice which guide actions that feed back to the environment. The selection process is affected by the internal state which includes motivations, tasks etc. The selection process can also influence the sensory organs (overt attention) or not (covert attention). **(B)** Under the EVA scheme, information from the environment is sub-sampled and transmitted to the brain, where it is used to make a decision of the target of behavior. The decision then feed back to enhance representations of signals that lead to target oriented behaviors, and to reduce representations of signals that lead to distracting behaviors (covert attention). In some cases the decision influences the sensory organs (overt attention).

The EVA builds on the above notion. An animal’s behavior is a continuous coordinated interaction with the environment, and attentional mechanisms control this interaction by dynamically allocating the orientation of the animal covertly and overtly. The main competition in attention is therefore not over limited internal processing resources. Instead, objects, events, features and locations in the external world compete for the privilege of being the target of behavior. This is the main link to Gibson’s ecological theory of perception. Perception, according to Gibson, exists to serve adaptive behavior. We perceive the world in accordance to the behavioral opportunities offered by the environment (Gibson, 2014). According to Gibson, it is misleading to think that the visual system first represents the world as detailed as possible and then processes this internal representation to identify what is out there (Barrett, 2011). The information about what is out there is available in the incoming input. What is needed is to use this information to make smart behavioral choices. In this sense, there is no point in first making a behavioral choice (shifting attention one way or the other) and then processing the relevant information. For making correct choices, it should be the other way around. That is, exhaust all the available information before making the choice to shift attention (Figure 2).

The EVA is not at all a unique theory in challenging the LBRV by rejecting the idea that attention evolved as a means to select events for detailed processing. Theories of attention for action promote late selection at the level of response, decision making or working memory (Deutsch and Deutsch, 1963; Bundesen, 1990; Deubel, 2014). However, most of the theories of late selection share with the LBRV the concept of competition for a limited internal resource. The limited resource according to late selection theories may be muscle activation (Deubel, 2014; Bulgheroni et al., 2017) or neural machinery for representations in a high-level working memory (Awh et al., 2006). The EVA, does not deny that the brain is physically limited, but it does not see physical limitations as the driving force behind selective attention. The competition between sensory events is imposed by the need to make the *correct* choice based on ambiguous information. The donkey, in the Buridan’s ass paradox, is indeed physically limited to walk only to one pile of hay. However, there are several other behavioral options. The donkey can randomly walk to a pile or can respond to both piles simultaneously, resulting with an average behavior. Both options are maladaptive. Evolution favors a donkey that can take every possible piece of information available, including internal information such as memory, context, internal biases and internal noise, to make a selection and use this selection to guide its behavior, that is, prioritize the subjectively favored pile and suppress the representation of the competing pile. The EVA, therefore, goes beyond many current views of attention by asserting that no physical tradeoffs are driving attention. It is in line with hypotheses that see attention as posed by the need to make accurate decisions for goal-directed behaviors with noisy sensory channels, such as the decision integration theory (Shaw, 1982), selection for goals proposition (Van Der Heijden and Bem, 1997a), attention for probabilistic computations (Eckstein, 2017) and the more recently published viewpoint about attention as an outcome of value-based decision making (Krauzlis et al., 2014, 2021). The EVA suggested here converges with these ideas from an evolutionary perspective.

## Comparative Studies of Attention Support the Ecological View of Attention

One main postulate of the EVA is that the selection of a target for response accompanied by suppression of responses to distractors (i.e., attention) is a very basic aspect of animal behavior. Thus, we predict that the spread of attention capabilities across the animal kingdom is an outcome of divergent evolution. In other words, we should be able to draw an evolutionary line connecting mechanisms of human attention to common ancestors of a very wide range of animals. Traditionally, the study of attention focused on humans, partly due to the ease of conducting behavioral experiments in humans. However, along the development of behavioral techniques in animals, evidence of “human-like” attention behaviors in animals is accumulating. The emerging picture is that attentional behaviors are remarkably similar across animals from far apart taxa (Mokeichev et al., 2010; Knudsen, 2018; Krauzlis et al., 2018). An example is the study by Sareen et al. (2011) that tested fruit flies in a typical Buridan’s ass paradox situation. When confronted with two moving bars on opposite sides, flies do not stay in the middle but choose one or the other side. However, this is not a random choice – the researchers could manipulate the choice by applying lateral visual cues. It is unlikely that the fly has any difficulty processing stimuli from both sides simultaneously, as each side is processed independently and in parallel in the corresponding optical lobe (Borst et al., 2020). The costs of using external cues to bias the selection of the fly are likely to be larger than taking a random decision strategy. Thus, selective attention in flies evolved to promote a behavior that is coordinated with the environment and the animal’s needs. If attention is a continuous trait in evolution, a similar evolutionary explanation should underlie humans’ selective attention.

Studies of bees demonstrated serial and parallel search strategies (Spaethe et al., 2006; Morawetz and Spaethe, 2012), selective visual attention (Paulk et al., 2014), and cueing effects (Eckstein et al., 2013). Not only are attention behaviors in insects reminiscent of human attention behaviors, but similarities have also been found in the underlying neural mechanisms. First, studies in bees and fruit flies show that brain networks for selective attention in insects are widespread, involving multiple brain regions (de Bivort and van Swinderen, 2016). Thus, like in mammals, target selection and suppression of distractors is a demanding computational task, occupying a substantial part of the brain’s activity. That large portions of even small brains take part in the stimulus selection process, suggests that brains evolved to limit information processing. Second, endogenous oscillations in the LFP of flies in the range of 20–50 Hz were associated with selective attention (van Swinderen, 2007), perhaps reminiscent of gamma oscillations that were linked with attention in humans and other vertebrates (Fries et al., 2001). Finally, and most striking, is the finding that dopamine levels in the mushroom bodies may regulate and maintain the cueing effect in the fly’s “brain” (Zhang et al., 2007); dopamine has been strongly linked with attention in humans and other species (Bahmani et al., 2019). These physiological similarities suggest that attention mechanisms are fundamentally diverging from common ancestors.

Comparative studies in vertebrates, from fish to primates, show strong behavioral, anatomical and physiological analogies to attention. To give a few examples of behavioral similarities: inhibition of return (IOR), the typical dynamical pattern of humans’ allocation of attention following attentional capture by a cue (Klein, 2000), has been shown in archer fish and barn owls (Gabay et al., 2013; Lev-Ari et al., 2020). Attentional capture and cueing effects on reaction time and accuracy in barn owls and chickens were shown to resemble human performance in similar tasks (Sridharan et al., 2014; Lev-Ari and Gutfreund, 2018). Pop-out perception and serial search in barn owls and archer fish also highly resemble human perception in visual search tasks (Ben-Tov et al., 2015; Orlowski et al., 2015). The above examples and more (Ingle, 1975; Krauzlis et al., 2018; Castro et al., 2020) support that attention is a common and related theme across vertebrates. However, can we find species that do not employ attention in their interactions with the environment? Chameleons are an interesting case study in this aspect. This reptilian species has a remarkable capability to move its two eyes independently of each other (Harkness, 1977; Ott, 2001). For example, one eye can maintain fixation on a potentially threatening object while the other continues to search the environment (Lev-Ari et al., 2016). It seems that the chameleon’s parallel, independent scanning of the two hemifields is a violation of attention principles. However, detailed measurements of the chameleons’ eye movements show that the two eyes are less independent than commonly thought; visual information from one eye dominates overall behavior, and importantly, when two competing prey items appear, at some point, the chameleon makes a decision to abandon one target and point both eyes toward the salient target (Katz et al., 2015). Thus, even in an animal that has the machinery for parallel tracking, attention eventually takes over. This stresses the ecological importance of making a behavioral choice and committing to this choice.

**FIGURE 3:**
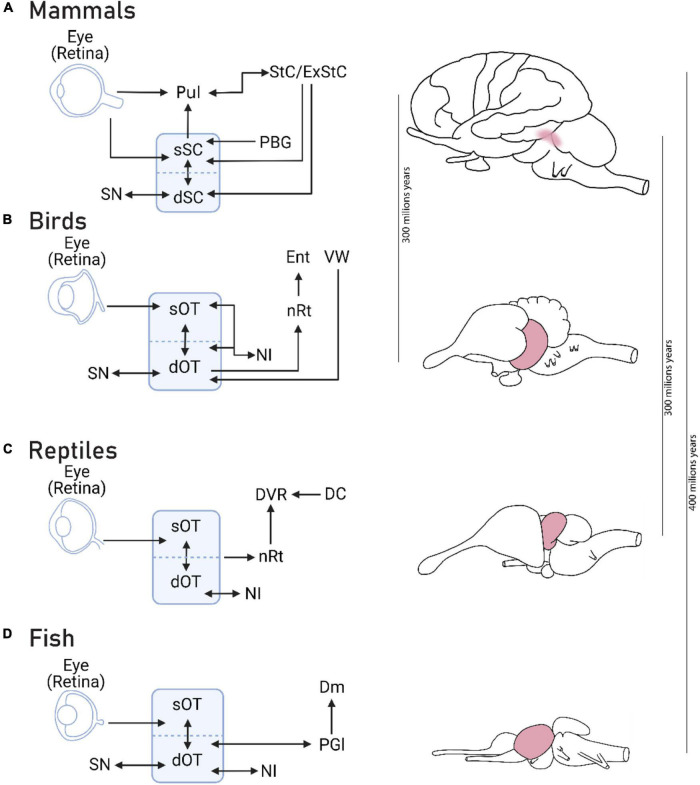
The basic visual system of vertebrates integrates and selects information. The basic visual system of vertebrates is centered on the optic tectum (superior colliculus in mammals), shown on the right, in gray, on illustrations of: a mammalian **(A)**, avian **(B)**, reptilian **(C),** and fish **(D)** brain. Across taxa, the OT integrates bottom-up and top-down visual information for the control of orienting responses. Reciprocal interactions with nucleus isthmi (NI) or Parabigeminal nucleus (PBG; in mammals) is a joint feature of OT across vertebrates, which contributes to stimulus selection (Knudsen, 2018). Dm: Dorsomedial pallial amygdala; DVR: Dorsal ventricular ridge; Ent: Entopallium; ExStC: Extrastriate cortex; NI: Nucleus isthmi; nRt: Nucleus rotundus; PBG: Parabigeminal nucleus; PGI: Lateral preglomerular nucleus; Pul: Pulvinar nucleus; SN: Substancia nigra; sOT/dOT: Superficial/deep Optic tectum; sSC/dSC: Superficial/deep Superior colliculus; StC: Striate cortex; VW: Visual wulst. Diagrams based on: mammals – Mueller (2012) and Krauzlis et al. (2013), fish – Soares et al. (2017); reptiles – Naumann and Laurent (2020); birds – Mysore and Knudsen (2011).

The optic tectum (OT), known as superior colliculus (SC) in mammals, is considered the primitive visual system in vertebrates (Kardamakis et al., 2015; Basso et al., 2021). Hence, inspection of the OT across vertebrates can provide clues as to the evolution of the visual system (Butler and Cotterill, 2006). Remarkably, the tectal retinotopic map in all species tested today is robustly context-dependent (Figure 3). That is, when two or more stimuli are presented simultaneously, the most salient stimulus is represented preferentially while responses to competing stimuli are mostly suppressed [reptiles (Saha et al., 2011); Aves (Mysore et al., 2010); fish (Volotsky et al., 2019); mammals (Pluta et al., 2011)]. Additionally, when stimuli are presented in succession, the deviants or surprising stimuli are represented preferentially (Reches and Gutfreund, 2008; Boehnke et al., 2011; Netser et al., 2011). This competitive property, both in space and time, is achieved through specialized and conserved networks, highly tailored for the task of selecting and highlighting the most behaviorally relevant stimulus at the expense of other stimuli (Reches and Gutfreund, 2008; Mysore et al., 2010; Mysore and Knudsen, 2011; Kardamakis et al., 2015; Garrido-Charad et al., 2018). The comparative observation that the basic visual map preferentially represents salient stimuli in distinct vertebrate species indicates the importance of stimulus selection in the evolution of the visual system (Fecteau and Munoz, 2006). Importantly, this ancient role of the tectal system in the control of selective attention was not lost in the course of human evolution. Lesions in the SC of macaque monkeys resulted in clear attentional neglect (Lovejoy and Krauzlis, 2009). In humans, imaging studies point to the involvement of the SC in attention (Katyal et al., 2010; Katyal and Ress, 2014). These observations suggest a common origin of attention across vertebrates. We speculate that stimulus selection mechanisms in the OT evolved to cope with conflicting stimuli and these mechanisms were preserved in evolution, manifested as selective attention in humans.

## How Can We Reconcile Current Neuronal and Behavioral Observations of Attention With the Ecological View of Attention?

The idea of a limited brain resource underlying selective attention has been driven by numerous psychophysical studies that demonstrate clear tradeoffs between the demands of the perceptual task and the perceptual suppression of distractors (Lavie, 2005). In difficult tasks, entitled high perceptual load, task-irrelevant stimuli are often missed, a phenomena called in-attentional blindness (Cartwright-Finch and Lavie, 2007). Importantly, when the perceptual load of the task is reduced (easy tasks), similar task-irrelevant stimuli are more likely to be detected and/or influence behavioral outcomes (Lavie, 2005; Forster and Lavie, 2008). Physiological measurements in a variety of cortical areas demonstrate an equivalent effect. For example, the BOLD signal in area V5 of unattended visual stimuli was modulated in accordance with the perceptual load of the engaged task. In difficult tasks, the signal was reduced compared to easy tasks (Rees et al., 1997). Similarly, firing rates in area V4 of macaque monkeys were modulated in accordance with the attended location and attentional load of the task (Reynolds and Desimone, 2003). This typical tradeoff in attention processes leads to the notion of competition over a limited resource that is allocated dynamically according to the demands of the task. A recent paper further supports this notion by showing that metabolic energy in the cortex during perceptual tasks is allocated away from unattended processing. Importantly, this effect was stronger in high-load tasks compared to low-load tasks (Bruckmaier et al., 2020). This suggests, in line with the LBRV, that energetic costs are limiting information processing, hence, attention evolved to allocate the resources, giving more to important stimuli at the expense of less important stimuli. However, these findings do not distinguish between cause and effect: is the behavioral and neuronal prioritization of the target a result of metabolic energy allocation in the cortex? Or is the pattern of energy allocation in the cortex the result of prioritization of the target? It is difficult to answer such chicken or egg questions experimentally. Addressing this question requires a theory. The first possibility fits the LBRV while the second the EVA.

Importantly, and often neglected, some of the resources allocated for the processing of the attended stimulus in high-load tasks is used to identify, separate, and suppress the responses to the distracting elements. It is, therefore, not obvious that the resources allocation is economically justified, as is required by the LBRV. The alternative explanation is that in difficult tasks, more resources are invested in suppressing irrelevant distractors, for behavioral reasons. Adaptive behavior is a delicate tradeoff between foraging for food and avoiding predators (Charalabidis et al., 2017; Eccard et al., 2020). The EVA asserts that attentional mechanisms dynamically control this balance according to the environmental conditions and behavioral states. When the targets of search are easily identified (low load), it is worthwhile to allow more attentional capture by distractors. However, in difficult conditions blockage of interference from distractors is needed. An animal searching for well-hidden, scarce food particles cannot allow itself to be easily distracted by task-irrelevant stimuli compared to an animal searching for salient, abundant food particles (Charalabidis et al., 2017). *Having an unlimited brain would not dispose this behavioral tradeoff because the sensory inputs themselves are limited and noisy* (van Beers et al., 2002*;* Lochmann and Deneve, 2011*).* Thus, the need to select a target and suppress distractors, i.e., selective attention, is likely present independent of the processing costs of the brain (Vincent, 2011; Eckstein et al., 2013).

The large body of research on attentional cueing has also been influential in the development of the LBRV (Posner, 1980; Pestilli and Carrasco, 2005). Generally, when two competing stimuli, a target and a distractor, are presented, the time to detect (reaction time; RT) the target when a cue predicts its location (valid cue) is shorter than the RT to the target when it is not associated with a cue (uncued). Whereas the RT to detect the target when the cue predicts a different location (invalid cue) is longer than the RT to detect the uncued target. In other words, a valid cue improves the behavioral response to a target and an invalid cue weakens the response (Carrasco, 2011). Why don’t we process both stimuli in parallel and to the fullest, independent of the cue? In principle, this should result in improved performance in the task. One answer is that the brain-limited capacity to process information in the task limits performance: we can only achieve full performance on one side and this comes with a cost on performance on the other side (Montagna et al., 2009). The EVA offers a different explanation. In natural environments, it is maladaptive to treat all incoming stimuli as equal because behavior is oriented and committed to a target. We evolved to select not because there is too much incoming information. On the contrary, the information entering the brain is very limited relative to the information in the environment, blurry and noisy (Deneve, 2012; Gibson, 2014). Hence, it is adaptive to use any cue as an additional source of information to improve the likelihood of correct selections (Vincent, 2015).

## Consequences of an Ecological View of Attention

First, an EVA emphasizes an animal’s interaction with its environment and the evolution of behavior in sculpting attention. It draws a connecting line from human attention to the basic ecological needs of an active organism. It calls for more comparative and general approaches in the study of attention in a wide variety of animal species. Second, the EVA removes the spotlight from limited capacity to the dynamic control of adaptive behavior. It thus links together fundamental cognitive phenomena that in most cases are studied separately. Under the EVA scheme, decision-making (Herman et al., 2018), categorization (Mysore and Kothari, 2020), optimal-inferences (Vincent, 2015), orienting (Bradley, 2009), habituation (Netser et al., 2011; Gutfreund, 2012), and surprise (Nelken, 2014) are linked with attention. The neural mechanisms of these cognitive phenomena may be more overlapping than is commonly believed. For example, the OT/SC has been linked in separate articles with decision making, attention, categorization, orienting and surprise (Muller et al., 2005; Boehnke and Munoz, 2008; Reches and Gutfreund, 2008; Lovejoy and Krauzlis, 2009; Netser et al., 2010; Boehnke et al., 2011; Mysore and Knudsen, 2012; Jun et al., 2021). Thus, the EVA calls for more interactions and mutual fertility between the different sub-fields of cognitive neuroscience. Finally, the EVA emphasizes attention as a mechanism for allocating behaviors, and thus attentional deficits are not sensory deficits or processing deficits but behavioral deficits. Under the EVA scheme, a subject suffering from an attentional deficit is not overwhelmed with sensory information but is overwhelmed with behavioral opportunities for which he has difficulty to choose and commit to the appropriate one. This way of thinking about attention may give rise to different ideas of how to address, diagnose and treat attentional deficits.

## Summary

Here, we ask why attention evolved and why it is so widespread among animal species. Our analysis suggests that attention is not selection for further detailed processing, but rather it is selection of a target for behavior. This notion agrees with the growing body of literature that highlights attention as a process sub-serving adaptive behavior (van der Heijden and Bem, 1997b; Krauzlis et al., 2014, 2021; Eckstein, 2017). We believe this shift in thought can be reconciled with what we observe about attention and, in addition, settle some of the conceptual difficulties of the LBRV. We identify two major bottlenecks in the path from the environment to the behavior. One is the vast amount of information in the environment, which is substantially sub-sampled by the sensory organs. The second is the reduction from multiple behavioral possibilities to one behavior that is most adaptive for the organism. These two bottlenecks are external to the brain, yet we argue that they form the dominant forces behind the evolution of attention. Thus, we suggest that attention is predominantly ecological: it implies how we sample the environment and how we interact with the environment.

## Data Availability Statement

The original contributions presented in the study are included in the article, further inquiries can be directed to the corresponding author.

## Author Contributions

TL-A, HB, and YG participated in writing the manuscript and creating the figures. YG coordinated the writing and edited the final version. All authors contributed to the article and approved the submitted version.

## Conflict of Interest

The authors declare that the research was conducted in the absence of any commercial or financial relationships that could be construed as a potential conflict of interest.

## Publisher’s Note

All claims expressed in this article are solely those of the authors and do not necessarily represent those of their affiliated organizations, or those of the publisher, the editors and the reviewers. Any product that may be evaluated in this article, or claim that may be made by its manufacturer, is not guaranteed or endorsed by the publisher.
